# Development of an Online Multilingual Educational Programme for Parents of Dual-Career Athletes: A Participatory Design

**DOI:** 10.3389/fpsyg.2022.855531

**Published:** 2022-07-22

**Authors:** Laura Capranica, Flavia Guidotti, Carlos Gonçalves, Laurence Blondel, Matteo Bovis, Rute Costa, Nadine Debois, Antonio Figueiredo, Ciaran MacDonncha, Viktorija Pecnikar-Oblak, Jean-Luc Patoret, Andrej Pišl, Eoin Rheinisch, Ana Rolo, Gary Ryan, Anne Templet, Antonio Tessitore, Giles Warrington, Mojca Doupona

**Affiliations:** ^1^Department of Movement, Human and Health Sciences, University of Rome “Foro Italico”, Rome, Italy; ^2^European Athlete as Student, European Athlete as Student (EAS) Network, Ghaxaq, Malta; ^3^Faculty of Sport Science and Physical Education, University of Coimbra, Coimbra, Portugal; ^4^National Institute of Sport, Expertise and Performance (INSEP), Paris, France; ^5^CONI, Rome, Italy; ^6^Ginàsio Clube Figueirense, Figueira da Foz, Portugal; ^7^Department of Physical Education and Sport Sciences, University of Limerick, Limerick, Ireland; ^8^Health Research Institute, University of Limerick, Limerick, Ireland; ^9^Department of Sport Sociology, Faculty of Sport, University of Ljubljana, Ljubljana, Slovenia; ^10^EUSA Institute, Ljubljana, Slovenia; ^11^Sport Ireland Institute, Dublin, Ireland

**Keywords:** dual career, online education, parenting athletes, student-athletes, experts

## Abstract

There is a need for educational support structures to empower parents in sustaining talented athletes pursuing academic and sports careers (e. g., dual career). The present work describes the participatory design used to develop a series of educational resources and the subsequent iterations used to evaluate the content of the EMPATIA online education programme for parents of dual-career athletes. Following an ethnographic approach, the project team (18 dual-career experts) engaged in four iterations (i.e., rounds) planned to develop (rounds 1 and 2) educational material from preliminary evidence (systematic literature review) and eminence (focus groups and concept mapping) knowledge and to evaluate the educational programme (round 3 and 4) engaging end users (*n* = 76) and other stakeholders (9 dual-career experts). The EMPATIA programme was developed and organized in four modules labeled after macro-aspects, parents could ask about dual career: “Why” (the definition and challenges of dual career for athletes and their parents), “What” (insights, guidelines, and suggestions on the parental role in support of student-athletes), “How” (practical advice on planning dual career at sports and academic levels, and post-sports careers), and “Where” (finding legal information or counseling on dual career). Despite overall positive evaluations of the programme, parents of dual-career athletes attributed higher scores with respect to those of dual-career experts. The participatory approach presented in this work enables developers to apply a systematic and multidisciplinary approach toward the creation of educational programmes for parents. The cooperation among dual-career researchers, experts from high-performance centers, Olympic bodies, sports clubs, and parents of elite student-athletes of different sports and nationalities created an educational programme suitable for end users to support parenting athletes in combining their sports and academic careers.

## Introduction

In Europe, 120,000 talented young European athletes are estimated to dedicate several years to achieving elite level in sports (European Commission, 2016). In general, a sports career starts at a young age, lasts into adulthood, and during its lifespan encompasses increasing volume, intensity, and organization of training and competitions organized at national and international levels. Parallel to sports careers, academic careers significantly increase the educational demands from mandatory education (e.g., primary and high school) to university. In considering that in the European Member States competitive sports are mainly structured at club level, talented and elite athletes are at risk of academic or sport drop outs when ducational and sport systems present cultural and organizational divergences (Aquilina and Henry, 2010; European Commission, 2016). In fact, a sports engagement of around 20 h per^.^week^−1^ and an academic commitment of around 30 h per.week^−1^ have been reported (Sheehan et al., 2018; Condello et al., 2019). To support the athletes' right to pursue their holistic development through the conciliation of their sports and academic careers, in 2007, the European white paper on sports introduced the term “dual career,” which is considered one of the priorities in European sports strategy and policy (European Commission, 2007, 2012, 2016, 2022; European Parliament, 2015, 2017, 2021).

From various perspectives, numerous studies have examined the factors that influence dual careers (Guidotti et al., 2015; Stambulova and Wylleman, 2019). Although, sportspersons are mainly responsible for their dual careers, several stakeholders have been identified as influencing the micro—(e.g., the individual athlete), the meso—(e.g., parents, peers, teachers/employers, coaches, sports managers), the macro—(e.g., sports clubs/federations, educational institutions, and labor market), and the policy (e.g., national and European governing bodies) levels of dual-career paths (European Commission, 2007, 2012; Guidotti et al., 2015; Capranica and Guidotti, 2016; Stambulova and Wylleman, 2019; Stambulova et al., 2021).

Actually, Member States adopt different approaches to dual careers, which determine a wide range of support measures and services also in relation to the sports-specific and education/work-specific needs of athletes (Aquilina and Henry, 2010; Capranica and Guidotti, 2016; European Commission, 2016). Independent from nationality and sport, at a personal level, elite athletes highlighted the crucial role of their parents, who provide emotional, motivational, instrumental, material, social, informational, and financial support (Condello et al., 2019). However, the personal experiences and opinions of parents regarding their supportive role of their dual-career children have been presented, and the complex parenting demands at individual and interindividual levels in relation to the family, sport, and academic environments have been systematized recently (Harwood and Knight, 2009, 2015; Harwood et al., 2010; Bean et al., 2014; Clarke and Harwood, 2014; Dorsch et al., 2017; Gjaka et al., 2021; Tessitore et al., 2021; Varga et al., 2021). To help parents facilitate relationships with dual-career stakeholders of family, sport, and educational entourages, the elaboration of a specific education programme appeared as a strategical necessity (Thrower et al., 2016; Capranica et al., 2018; Gjaka et al., 2021). With the support of the European ERASMUS+ Programme (https://erasmus-plus.ec.europa.eu), a consortium of 10 universities and sports institutions from six Member States (Ireland, Italy, France, Malta, Portugal, and Slovenia) having a consolidated relationship at national and European levels in dual career aimed to structure an Education Model for Parents of Athletes in Academics (EMPATIA, 590437-EPP-1-2017-1-SI-SPO-SCP).

To understand the values, beliefs, behaviors, and needs of parents of dual-career athletes from different countries and sports environments, from an epistemological point of view, the adoption of an ethnographic stance appears plausible to gain a comprehensive understanding of the group members' perceptions, as they shaped by their social and cultural settings. Therefore, the multidisciplinary EMPATIA team used an ethnographic research approach, which helps to describe a group or culture, strongly relying on personal experiences to make additional decisions about which approaches are appropriate for the situation at hand (Genzuk, 2003). Thus, a participatory approach was deemed a key feature for ensuring an active engagement of parents in the developing process of a standalone digital educational resource, self-directed in nature, freely and openly available in the form of reusable learning objects, and based on the small, independent, reusable, aggregation-ready educational units (McGreal, 2004). Indeed, a participatory design could ensure that a programme having usable, simple, and intelligible contents, aligned to end-users' needs, could ultimately empower the people involved (Ferguson et al., 2018; Hansen et al., 2019). According to the ethnographic research approach, to gather a comprehensive understanding of parenting dual-career athletes through the key aspects, their structure, and interrelationships, a variety of information gathered from different perspectives is crucial (Genzuk, 2003). First, an analysis of documents encompassed a systematic literature review, which highlighted fragmented information presenting a two-level construct of individual and interindividual aspects of parental support strategies of dual-career athletes (Tessitore et al., 2021). Second, to collect a variety of information on the personal experience of parents from different perspectives, the research included 12 focus groups involving a total of 115 parents of elite dual-career athletes, who discussed themes related to the athletes' needs, sports environment, academic environment, dual-career-related policies and services, and educational methods for parenting dual-career athletes (Gjaka et al., 2021). Thus, the parents condensed into 80 statements their perceived needs and opinions on the most relevant content of an educational programme for parenting dual-career athletes, mainly related to the parental support athletes' needs, the parents' relationships with the sports and academic entourages of the athletes, the parents' need of information on dual-career policies/services, and educational resources for parenting dual-career athletes. Finally, to uncover the cultural perspectives on which parenting dual-career athletes is based, the 80 statements were further integrated and visually and numerically represented in a concept mapping, which resulted in the composite thinking of 334 groups of parents of elite athletes as students around the complex dual-career phenomenon (Varga et al., 2021). The findings presented a five-cluster framework on the parents' roles, needs, and awareness to support athletes; requirements for effective planning of dual-career pathway; educational opportunity; policy and provision for dual career; and athletes' lifestyle and self-management.

Thus, the main objective of this paper was to describe the participatory design that is used to develop a series of educational resources and the subsequent iterations that are used to provide formative feedback on the content of the EMPATIA education programme for parents of dual-career athletes as a part of the verification process. It was hypothesized that core information and key priorities could lead to relevant and high-quality educational materials aligned to end-users' needs and a positive and structured feedback from a selected expert panel (Genzuk, 2003; Hsu and Sandford, 2007; Diamond et al., 2014).

## Methods

### Experimental Approach to the Study

Based on the sound triangulation of the different data collected in the background phase presented in the introduction (Gjaka et al., 2021; Tessitore et al., 2021; Varga et al., 2021), during a 2-year period, four iterations (i.e., rounds) were considered necessary to develop the EMPATIA education programme through qualitative approaches and to reach an agreed degree of consensus through qualitative and quantitative methods (Genzuk, 2003; Hsu and Sandford, 2007). In particular, the EMPATIA team was entitled to select wisely the key informants from the parents' perspectives and to use them carefully for developing a manageable online educational programme, whereas the parents of athletes as students and the dual-career experts represented the insiders to cross-evaluate that the researchers organized effectively educational material without omitting to include the essential aspects for empowering parents of dual-career athletes (Genzuk, 2003).

**Figure 1:**
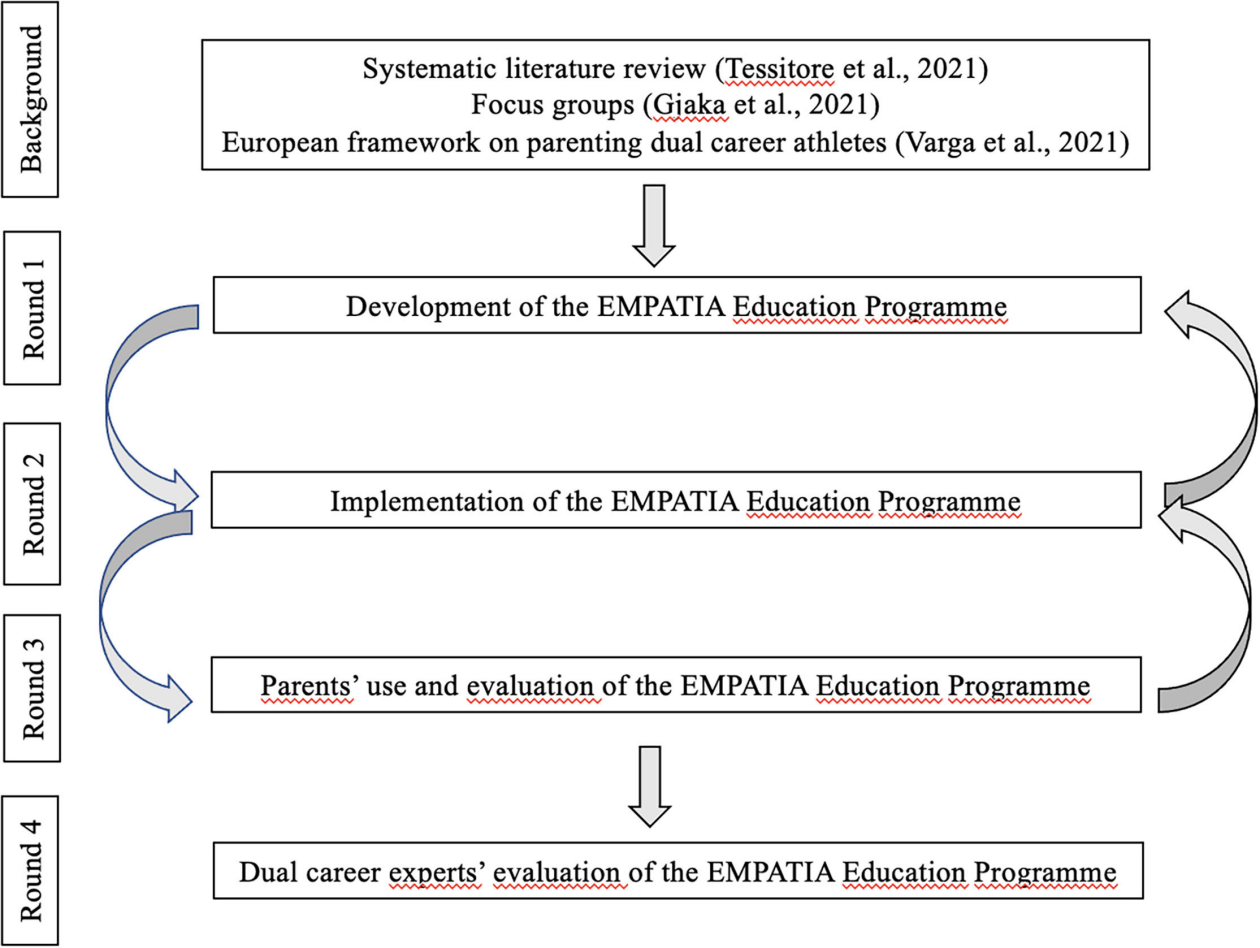
Schematic representation of the four rounds of the development of an online multilingual EMPATIA education programme for parents of dual-career athletes, illustrating the constant back-and-forth circulation of opinions, suggestions, and feedback, between the experts and the end users (i.e., the athletes' parents).

As schematically presented in Figure 1, rounds 1 and 2 were dedicated to the developmental phase, whereas during the second year, a consensus was reached through rounds 3 and 4.

#### Round 1

A total of two face-to-face meetings among the EMPATIA team members were organized to identify the most relevant inputs deriving from the five clusters (i.e., policy and provision for dual career; parent/guardian roles, needs and awareness to support athletes' health, wellbeing, sports, and education; athletes' lifestyle and self-management; requirements for effective planning of DC pathway; and educational opportunities) of the EMPATIA framework on parenting dual-career athletes (Varga et al., 2021). This interpretive process required a consensus on three challenges: first, to move from the logic deriving from a concept mapping to a conceptual logic of interconnected constructs that could respond to the educational needs of parents; second, to build coherent pedagogical units, conceptually coherent, to be used in sequence or separately that fit the constraints of an internet page, maintaining an appealing and user-friendly structure; third, to ensure a viable, interactive internet structure for allowing a separate and easy search of specific topics of interest.

#### Round 2

The EMPATIA team members established the preliminary priorities among educational items and agreed the learning goals encompassed the following pedagogical components: (i) the presentation of the concept or procedure to support the learning objectives; (ii) an activity for the learner to engage with the content; (iii) self-assessment to test mastery of the content; and (iv) links to further resources to reinforce the learning. Then, a consensus on two distinct phases was required: First, the prototype is developed to obtain a full specification of the future programme. Second, the formal educational programme is provided in English, French, Italian, Portuguese, and Slovenian.

#### Round 3

National workshops were organized to present the EMPATIA education platform to parents of dual-career athletes. After allowing the participants to navigate individually the programme, an evaluation questionnaire including ratings and open judgments was administered. This round provided an opportunity for clarifications on the relative importance and comprehensibility of the educational items, as well as provided an opportunity to revise the educational programme, if needed.

#### Round 4

In the final round, a workshop with international dual-career experts was organized to present the EMPATIA education platform. After allowing the participants to navigate the programme individually, an evaluation questionnaire including ratings and open judgments was administered. This round provided a final opportunity to revise the educational programme, if needed.

### Participants

Three groups of well-qualified experts were identified: (1) 18 dual-career experts from three universities with sports science programmes, two high-performance centers, Olympic bodies, sports clubs, an internet designer, and all members of the EMPATIA team. This group was entitled to rounds 1 and 2; (2) a sample of 79 parents (F = 47%, M = 53%; education: university = 69%, high school = 31%) of dual-career athletes (range: 14–24 years) competing in team (72%) and individual (28%) sports at national (highest championships: 53%) and international (European and World competitions; 47%) levels was drawn from the national databases of parents who previously contributed to the development of the EMPATIA framework on parenting dual-career athletes (Varga et al., 2021).

This group was engaged in round 3. All potential participants received a notification email requesting to contribute to the evaluation of the EMPATIA education programme based on the results of the previous concept mapping consultation of the EMPATIA framework, the time required and the nature of the online evaluation exercise, and information that their participation was entirely voluntary, that they could withdraw at any time without providing a reason, that their consent to participate was implied by submitting the online survey, and that all responses were anonymous and confidential in nature; and (3) nine dual-career expert delegates of European institutions members of the European dual-career network (EAS; www.dualcareer.eu). This group was engaged in round 4. All potential participants were invited to the 2021 Annual Conference of the European Athlete as Student, and they were informed about the EMPATIA project and the development of the educational platform, the time required and the nature of the online evaluation exercise, and information that their participation was entirely voluntary, that they could withdraw at any time without providing a reason, that their consent to participate was implied by submitting the online survey, and that all responses were anonymous and confidential in nature.

### The Instrument

Due to a lack of a validated scale for the assessment of an educational programme for parents of dual-career athletes, an electronic and multilingual (i.e., English, French, Italian, Portuguese, and Slovenian) evaluation questionnaire was developed (Appendix A) to allow a time and geographic flexibilities, as well as multimedia and self-administration modalities (Callegaro et al., 2015). From an applied point of view, this self-report measurement instrument has been developed as probes and follow-up questions (Genzuk, 2003). The questionnaire included a preliminary section to gather socio-demographic information from the sample, encompassing the sport practiced by the child, and the country, sex, and educational level of the parent. Then, 14 questions were developed to gather the parents' feedback on their first personal impression of the EMPATIA education programme (Q1–Q5), their perceived relevance of the educational modules for parental support of dual-career athletes, the management of the child's sports and academic environments and the child's dual-career transitions (Q6–Q10), their opinions regarding the texting and the visual structure, their overall evaluation score (Q11–Q13), and suggestions for further improvements (Q14). For the dual-career experts' evaluation, only the questions related to the quality of the programme (Q1–Q5, Q12–Q14) were considered.

To collect data on a large sample of a heterogeneous population, close-ended questions were chosen (e.g., single or multiple response checklist type) even though respondents were also allowed to elaborate further on their answers.

### Statistical Analysis

Data were analyzed using the Statistical Package for the Social Science, version 24.0 (SPSS Inc., Chicago Illinois). Descriptive statistics was applied, and the frequency of occurrence (expressed in absolute values or percentages) was calculated for the questions for which a single response or multiple responses were allowed. Except for neutral responses, data reduction has been conducted by collapsing very positive and positive responses, as well as negative and very negative responses. Then, the chi-square tests were applied to verify the distribution of answers between parents and dual-career experts, with a level of statistical significance set at *p* < 0.05 for all computations. Finally, the sum of positive or neutral responses >70% was considered a cutoff for agreement.

## Results

### Developmental Phase

During the initial developmental phase, an agreement defined the overall structure of the educational programme for guiding the users in their search and interpretation of information. It was deemed crucial to address the demands of dual careers and the role of parents, and to suggest resources for parents in search of supplementary information (videos, papers, books, online sites, etc.). Then, the best-ranked statements/items included in the concept mapping of the EMPATIA framework were organized in four modules labeled after macro-aspects, and parents could ask about dual careers: “Why,” “What,” “How,” and “Where.” Finally, the online platform's planning and design were approved to ensure an effective data indexing to enhance the search operation. The implementation phase tested the developed system regarding the expected results and the defect-free requirements of the system. Furthermore, an agreement was reached regarding the content of the four modules for quality education. In particular, the “Why” module encompassed the definition of dual career and its structure, the importance of sports and educational careers, the challenges of dual careers for athletes and their parents, information on European dual-career guidelines, and policies in place in the Member States. Furthermore, video recordings of athletes, coaches, dual-career policy officers, and parents were provided. The “What” module offered some insights, guidelines, and suggestions on the parental role to support athletes in balancing the dual demand of maintaining a sporting and academic career pathway and potential sources of stress for both the athletes and the whole family. The “How” module included practical advice on planning dual careers, lifestyles, self-management, and dual-career transitions to new environments, at sports and academic levels, and to post-sports careers. Finally, the “Where” module was meant to assist parents in finding legal information or counseling about their rights and doubts about dual careers. While offering mainly the information on specific legislation or provisional services and resources for France, Ireland, Italy, Portugal, and Slovenia, it also extended the information on dual careers in Europe, official European documents and studies, and suggested further readings.

The educational content was developed in English and translated into French, Italian, Portuguese, and Slovenian by two native experts in each language, who independently performed the forward translation and agreed on a combined version. To avoid inaccuracies in producing a conceptually and semantic equivalent translation, an English reviewer performed a backward translation with a blind procedure (Su and Parham, 2002).

### Evaluation Phases

All the evaluations of both the parents and the dual-career experts' groups reached a consensus, with significantly higher (97.5 ± 6.8%) positive evaluations for the parents (89.3 ± 10.9%) with respect to those of their dual-career counterparts (Table 1). For both groups, no negative response emerged for the first impression of the programme and the relevance of the four education modules, which received very high positive responses, especially from parents (range 95–98%). To note, a lack of difference between groups emerged only for the relevance of the module WHY. While the majority of parents considered the clarity and simplicity of the text very positive (range: 93–94%) or adequate (range 3–5%), the provided information received lower scores (positive: 49%; adequate: 26%). Interesting, only for this issue, the sum of positive and neutral evaluations of dual-career experts was higher values (86%) with respect to those of parents (76%). While parents attributed high positive evaluations (91–99%) to the visual structure of the programme considered very easy to navigate and well-structured, the dual-career experts attributed their lowest positive scores (51%) and the highest frequency of negative ones (14–29%). Nonetheless, the overall score of the EMPATIA education programme was positive (parents: 95%; dual-career experts: 71%).

**Table 1:** Frequency of occurrence (%) of negative, neutral, and positive evaluations of the EMPATIA programme and statistical comparisons (*p* < 0.05) between parents of dual-career athletes and European dual-career experts.

		**Parents**	**Experts**	
**Questions**		**%**	**%**	***P***
**First impression of the EMPATIA programme**				
	Negative	0	0	<0.001
	Neutral	0	18	
	Positive	100	82	
**Relevance of the module “HOW”**				
	Negative	0	0	0.001
	Neutral	4	18	
	Positive	96	82	
**Relevance of the module “WHERE”**				
	Negative	0	0	0.009
	Neutral	2	11	
	Positive	98	89	
**Relevance of the module “WHAT”**				
	Negative	0	0	<0.001
	Neutral	2	22	
	Positive	98	78	
**Relevance of the module “WHY”**				
	Negative	0	0	0.083
	Neutral	5	11	
	Positive	95	89	
**Text of the EMPATIA programme**				
Easy to understand	Negative	3	14	<0.001
	Neutral	3	29	
	Positive	94	57	
Simple language	Negative	1	14	<0.001
	Neutral	5	29	
	Positive	94	57	
Quantity of information	Negative	24	14	<0.001
	Neutral	27	43	
	Positive	49	43	
**Visual structure of the EMPATIA programme**				
Well-structured	Negative	0	29	<0.001
	Neutral	5	14	
	Positive	95	57	
Easy to navigate	Negative	1	29	<0.001
	Neutral	8	14	
	Positive	91	57	
Clear structure	Negative	1	14	<0.001
	Neutral	8	29	
	Positive	91	57	
**Overall score of the EMPATIA programme**				
	Negative	0	14	<0.001
	Positive	5	14	
	Neutral	95	71	

Also, the evaluations of the parents' perceived relevance of the educational modules for parental support of dual-career athletes, the management of the child's sport and academic environments, and the child's dual-career transitions resulted in high positive evaluations regarding the information for understanding the parental role in dual career (94%) to help the athletes managing their time (89%), financial issues (88%), healthy lifestyles (93%), and nutrition (84%), and preventing doping (84%), the latter presenting one negative response. The EMPATIA education programme was perceived as very helpful for the parental role in supporting the athletes in their sports environment by means of effective strategies (88%), managing difficult conversations (86%), recognizing bad (81%), and friendly (86%) sports environments, and balancing sports commitments (95%). Similarly, very positive feedback emerged relative to the academic environment, with high percentages for effective strategies (83%), managing difficult conversations (84%), recognizing bad (88%), and friendly (90%) academic environments, and in balancing academic commitments (93%). Finally, the EMPATIA programme was considered helpful for the parental role (89%) in support of athletes during academic (86%) and sports (86%) transitions, to adapt to transitions (86%) and relocations (80%), and during injuries and post-sports careers.

## Discussion

To improve the usability and satisfaction of sound educational programmes for the implementation of competencies of end users, European research funding bodies encourage capacity building based on sound theory and design principles, as well as end users and stakeholders viewed as co-constructors of the educational tools at the core of the development and evaluation phases (European Commission, 2022). Furthermore, the adopted ethnographic research approach allowed a comprehensive understanding of European parents' needs as shaped by their national sociocultural dual-career settings and different sports environments (Genzuk, 2003). In line with the above principles, the main findings of this study substantiated the value of a participatory development process of the online multilingual EMPATIA education programme based on evidence and eminence knowledge on the educational needs of parents of dual-career athletes, which was positively evaluated through a process encompassing stakeholder consultations (Capranica et al., 2018; Hansen et al., 2019; Gjaka et al., 2021; Tessitore et al., 2021; Varga et al., 2021). Interesting to note, the parents of dual-career athletes (e.g., end users) appreciated very much the overall quality of the programme (i.e., Q1–Q5 and Q11–Q14), whereas dual-career experts tended to be positive, yet more moderately. This partial mismatch might highlight the parents' feelings to be considered relevant actors in need of information on dual careers, a relevant phenomenon heavily conditioning the life of their entire family. It is also possible to speculate that parenting dual-career athletes is a relatively novel issue also for dual-career experts, who might give the parental support of athletes as granted. Indeed, there is a need to establish a valuable dual-career alliance also including parents to facilitate sharing of the information and experiences on specific dual-career demands, development programmes, and processes (Gjaka et al., 2021; Tessitore et al., 2021).

The positive evaluations of parents mirror their appreciation of information strictly related to the priorities they highlighted during the collective conceptualization of parental support of dual-career athletes (Varga et al., 2021). According to the principles of participative approaches (Hansen et al., 2019), the parents were required to evaluate the aspects related to the programme's outcomes and impact, especially those related to their personal gains in supporting the dual career of their talented child. Overall, the parents perceived the educational content of the EMPATIA programme as helpful at the individual level not only for clarifying their role in supporting dual careers but also in helping the athletes manage healthy lifestyles (range 83–94%). Starting from a relevant responsibility in providing motivation, encouragement, and support toward the holistic development of their talented children, parents need to be properly informed on short- and long-term plans for sound educational and sports decisions, which could have immediate and/or longer-term implications for their children and their family. In fact, intergenerational transfer of skills, knowledge, and social ability needs time and specific competencies, so parents have to be aware and ready to fine-tune their support in relation to the different challenging aspects of dual-career pathways during the developmental years of the athletes. Indeed, dual careers have to be considered as a lifelong process aiming to ensure the wellbeing of student-athletes, which includes their health-related needs to achieve and maintain an optimal mental and physical condition, to prevent and manage injuries and burnouts, and to avoid risky behaviors reported in athletes, such as unhealthy dietary habits, illegal supplement, alcohol, drugs, bullying, depression, gambling, match-fixing, violence, and sexual harassment (Aunola et al., 2018; Knight et al., 2018; Sorkkila et al., 2020; Gjaka et al., 2021; Stambulova et al., 2021).

In addressing the emphasis, stress, and perspectives on sports and/or academic achievements, the parents considered the EMPATIA education programme also beneficial for their interindividual relationship with the athletes and other dual-career stakeholders at family, academic, and sports levels (range 81–90%). Actually, parents share the responsibility of accompanying talented athletes with a multiplicity of stakeholders (e.g., the partner, other children, peers, teachers/employers, coaches, sports managers, etc.) having direct and strong influences on dual-career paths (Capranica and Guidotti, 2016). Despite the differences in national policies, services, and sociocultural contexts at sports and academic levels have been reported to challenge especially the interindividual relationships of parents, the information gathered based on cross-national and cross-sports research on the parents' views of their actual educational needs proved to be useful for a fine identification of relevant information to be included in the EMPATIA education units (Gjaka et al., 2021; Tessitore et al., 2021; Varga et al., 2021). In particular, the parents valued the useful information on the strategies and quality of communication to manage difficult conversations and conflicts in the family, sports, and academic environments and in uncovering signs that characterize negative and positive academic and sports environments. Indeed, good and regular parents-athlete-teacher/coach communication is deemed crucial to facilitate a successful dual-career alliance as well as to foster effective parental interventions at academic and/or sports levels, which could also lessen potential stress for parents. Parents educated in supporting dual careers could be valuable human resources for both sports organizations and educational bodies to ensure a continuous communication regarding the progress of the athlete's holistic development, especially when there is a need to negotiate flexibility, tutoring, and services and to design sound educational and sport short- and long-term wellbeing programmes. In going beyond the mastery of sport-specific skills as the athlete ages, the mentorship role of a coach is especially relevant during transitions to new environments requiring a variety of contextual changes that include people, culture, interactions, and social settings (Cosh and Tully, 2014; Holl and Burnett, 2014; Knight et al., 2016; Condello et al., 2019; Owiti and Hauw, 2021). Thus, positive interaction with coaches for athletic and academic achievements is required to encourage athletes enduring education requirements as well. A positive interaction between parents and academic staff is also crucial not only to help athletes manage major issues related to balancing timetables, tiredness, feeling of school belonging, goal settings, and personal development to prepare for post-sporting careers but also to foster a leadership role of student-athletes as role models in supporting active and healthy lifestyles promotion in the academic population, which could benefit from the close connection between learning abilities and physical movement or sports activities (O'Neill et al., 2017; Hills et al., 2019; Tomporowski and Pesce, 2019; López-Flores et al., 2021; Pesce et al., 2021). In fact, in considering education programmes as a capacity-building process, it is crucial to recognize and share the accumulated and valuable knowledge and experiences of the athletes' families to build impactful and sustainable collaborative partnerships.

## Conclusion

Although sports organizations and academic institutions are mainly responsible for their dual-career policies, if any, in the last decade, the European Parliament and Commission highly promoted the establishment and management of friendly environments for student-athletes through the sports call of the ERASMUS+ programme funding topic “Promote education in and through sport with special focus on skills development, as well support the implementation of the EU Guidelines on Dual Careers of Athletes” (European Commission, 2012, 2022; European Parliament, 2015, 2017). Despite these efforts in fostering a European dual-career discourse, the development of dual-career environments for the entire dual-career pathway (from school-age to university and vocation) in Member States is still limited, particularly in relation to the empowerment of parents of athletes (Guidotti et al., 2015; Capranica and Guidotti, 2016; Stambulova and Wylleman, 2019; López-Flores et al., 2021; Morris et al., 2021; Tessitore et al., 2021). The present work was the first EU-funded collaborative partnership addressing the issues of sustaining high school and university student-athletes by educating the supporting capabilities of their parents (Capranica et al., 2018). However, there is a need for further investigations to provide empirical validation of an assessment tool for the lifelong learning competencies of parents of dual-career athletes. Moreover, future studies are needed to investigate sports and academic bodies developing and supporting inclusive schemes and initiatives to comply with the European Guidelines, also including communication or formal arrangements with parents (European Commission, 2012). To bridge the gap between dual-career policies and practice, the online multilingual EMPATIA education programme can be considered a valuable resource and good practice for encouraging further initiatives based on a participatory and a user-centered design approach to describe implementation context and apply that information. In illuminating and understanding the needs of the dual-career actors, and in translating the information on actual needs in a free online education, the EMPATIA education programme could generate gains at individual levels and enhance the implementation of the European dual-career culture.

## Data Availability Statement

The raw data supporting the conclusions of this article will be made available by the authors, without undue reservation.

## Ethics Statement

The studies involving human participants were reviewed and approved by Research Ethics Committee of the University of Ljubljana of the EMPATIA project (9:2018). The patients/participants provided their written informed consent to participate in this study.

## Author Contributions

All authors listed have made a substantial, direct, and intellectual contribution to the work and approved it for publication.

## Funding

This work was supported by the European Commission under the Erasmus + Programme (number 590437-EPP-1-2017-1-SI-SPO-SCP).

## Conflict of Interest

The authors declare that the research was conducted in the absence of any commercial or financial relationships that could be construed as a potential conflict of interest.

## Publisher's Note

All claims expressed in this article are solely those of the authors and do not necessarily represent those of their affiliated organizations, or those of the publisher, the editors and the reviewers. Any product that may be evaluated in this article, or claim that may be made by its manufacturer, is not guaranteed or endorsed by the publisher.
