# Supplementary material for: Development of an Online Multilingual Educational Programme for Parents of Dual-Career Athletes: A Participatory Design
Source: Front Psychol. 2022 Jul 22;13:855531. doi: 10.3389/fpsyg.2022.855531 (PMC9354488; doi:10.3389/fpsyg.2022.855531)
Supplement: Supplementary file 1 [file Data_Sheet_1.pdf]

Appendix A: Questionnaire for Parents

Thank you for participating in the EMPATIA project. The purpose of this evaluation questionnaire (which will take no more than 15 minutes to complete) is to establish your views and opinions on how satisfied you are with EMPATIA educational materials developed. Please, fill out the questionnaire and mark the responses which best reflect your views. There are 14 questions in total and we would greatly appreciate it if you could answer all questions. In a number of the questions, you only need to mark one answer. Where you have the opportunity to provide more than one answer to a question this is indicated. There are no correct or incorrect answers. For a number of questions a comments box is provided to allow you to provide additional comments if you wish to. The questionnaire is anonymous and all responses will be kept completely confidential.

Part A: Socio-demographic characteristics

The sport discipline of your child: ______________________________

Your Nationality: ____________________

Your Gender:

male

female

Your education.Indicate the highest qualification. (tick the appropriate box)

No school qualification

Primary school qualification

Professional school qualification

High school qualification

University bachelor’s degree

University master’s degree

University PhD degree

Other (please specify).________________________________

Part B: the EMPATIA education programme

1. **How would you rate your first personal impression of the EMPATIA educational program?**

- Very negative
- Negative
- Neither positive or negative
- Positive
- Very positive
- Please provide additional comments on your first experiences of the EMPATIA Education Program (either positive or areas for improvement):_______________

2. **How relevant do you perceive the module ‘HOW’ was for supporting your child’s dual career?** *HOW you can help plan a dual career (time and sports environment, lifestyle, etc.).*

   (choose **one** of the following answers by ticking the relevant box):

- Highly relevant
- Relevant
- Neutral

- Limited relevance
- Not relevant at all
- Additional comments (optional):____

3. **How relevant do you perceive the module 'WHERE' was for the field of dual career?** *WHERE in the country you can find programs that support and encourage dual careers?*

   choose **one** of the following answers by ticking the relevant box):

- Highly relevant
- Relevant
- Neutral
- Limited relevance
- Not relevant at all
- Additional comments (optional):____

4. **How relevant do you perceive module 'WHAT' was for your personal dual career knowledge?** *WHAT is your role in supporting a child's dual career.*

   (choose **one** of the following answers by ticking the relevant box)

- Highly relevant
- Relevant
- Neutral
- Limited relevance
- Not relevant at all
- Additional comments (optional):____

5. **How relevant do you perceive module 'WHY' was for your personal dual career knowledge?** *WHY education is important for an athlete; WHY you as a parent/guardian should know the guidelines for a dual career.*

   (choose **one** of the following answers by ticking the relevant box):

- Highly relevant
- Relevant
- Neutral
- Limited relevance
- Not relevant at all
- Additional comments (optional):________________________________

6. **Does the EMPATIA education programme help your parental role in managing better the sport environment?**

   On a 5-point scale indicate your agreement (1 = I completely disagree; 2 = I partially disagree; 3 = I am neutral; 4 = I partially agree; 5 = I completely agree)

- I know better how to support my child balancing her/his sports commitments
- I know better to help the athlete creating a friendly dual career sport environment
- I am more aware of what a "bad" dual career sport environment looks like
- I am more confident in supporting my child having "difficult conversations" in the sports environment
- I am more aware of dual career policies/strategies in the sport environment in my country

7. **Does the EMPATIA education programme help your parental role in managing better the academic environment?**

On a 5-point scale indicate your agreement (1 = I completely disagree; 2 = I partially disagree; 3 = I am neutral; 4 = I partially agree; 5 = I completely agree)

- I know better how to support my child balancing her/his academic commitments
- I know better to help the athlete creating a friendly dual career academic environment
- I am more aware of what a "Bad" dual career academic environment looks like
- I am better able to support my child having "difficult conversations" in the academic environment
- I am more aware of dual career policies/strategies in the academic environment in my country

8. Does the EMPATIA education programme help you supporting your child's self-management?

On a 5-point scale indicate your agreement (1 = I completely disagree; 2 = I partially disagree; 3 = I am neutral; 4 = I partially agree; 5 = I completely agree)

- I better understand my role in supporting my child self-managing his/her dual career commitments
- I can better support my child managing his/her time
- I have more information for helping my child self-managing his/her financial budget
- I have more information for supporting the health and wellbeing of my child
- I have more information for supporting the healthy eating of my child
- I know more about the role a parent in raising the child's awareness of anti-doping issues

9. **Does the EMPATIA education programme help you supporting your child's dual career transitions?**

   On a 5-point scale indicate your agreement (1 = I completely disagree; 2 = I partially disagree; 3 = I am neutral; 4 = I partially agree; 5 = I completely agree)

- I know more about the transitions of my child from school to university
- I know more about the transitions of my child from youth to elite sport
- I know more about how I can help my child adapting to his/her transitions in the course of his/her dual careers
- I better understand the transitions of my parenting role as supporter of my child during his/her dual career
- I know more about the transitions of my child when relocating for sport and/or academic purposes
- I know more about the transitions of my child from healthy to injured conditions
- I know more about the transitions of my child from competitive sport to sport retirement

10. **Indicate your opinion regarding the text of the EMPATIA education programme**

    On a 5-point scale indicate your agreement (1 = I completely disagree; 2 = I partially disagree; 3 = I am neutral; 4 = I partially agree; 5 = I completely agree)

- Easy to understand
- Simple language
- Too much information

11. **Indicate your opinion regarding the visual structure of the EMPATIA learning programme**

    On a 5-point scale indicate your agreement (1 = I completely disagree; 2 = I partially disagree; 3 = I am neutral; 4 = I partially agree; 5 = I completely agree)

- Well structured
- Easy to navigate
- Clear

12. **On a five point scale indicate your overall score regarding the EMPATIA education programme**

    (choose **one** of the following answers by ticking the relevant box):

- Poor
- Needs improvement
- Average
- Good
- Very Good

13. **Please provide us your suggestions for further improving the applicability of the EMPATIA education programme:________________________________**

Thank you for completing the questionnaire

The EMPATIA Research Project Team
